# Enduring extreme climate: Effects of severe drought on *Triatoma brasiliensis* populations in wild and man-made habitats of the Caatinga

**DOI:** 10.1371/journal.pntd.0007766

**Published:** 2019-10-10

**Authors:** Antonia C. Ribeiro, Otília Sarquis, Marli M. Lima, Fernando Abad-Franch

**Affiliations:** 1 Laboratório de Ecoepidemiologia da Doença de Chagas, Instituto Oswaldo Cruz–Fiocruz, Rio de Janeiro, Rio de Janeiro, Brazil; 2 Grupo Triatomíneos, Instituto René Rachou–Fiocruz, Belo Horizonte, Minas Gerais, Brazil; 3 Núcleo de Medicina Tropical, Faculdade de Medicina, Universidade de Brasília, Brasília, Distrito Federal, Brazil; RTI International, UNITED STATES

## Abstract

**Background:**

*Triatoma brasiliensis*, a triatomine-bug vector of Chagas disease, evolved in the semiarid Caatinga, where it occupies rocky outcrops, shrubby cacti, and human dwellings. Dwellings and rocks are considered high-quality microhabitats for this saxicolous species, whereas cacti probably represent secondary, lower-quality microhabitats. This ‘microhabitat-quality hierarchy’ hypothesis predicts that *T*. *brasiliensis* populations occupying dwellings or rocks should endure harsh environmental conditions better than their cactus-living relatives.

**Methods/Findings:**

We tested this prediction by comparing *T*. *brasiliensis* infestation (proportion of microhabitats with bugs), density (bugs per microhabitat), and crowding (bugs per infested microhabitat) in dwellings, rocks, and cacti sampled before and during the extreme drought that ravaged the Caatinga in 2012–2016. We used random-intercepts generalized linear mixed models to account for microhabitat spatial clustering and for variations in bug-catch effort; we assessed model performance and computed model-averaged effect estimates using Akaike’s information criterion. Pre-drought infestation was similar across microhabitat types; during the drought, infestation remained stable in dwellings and rocks but dropped in cacti. Pre-drought bug density declined from dwellings to rocks to cacti; an additional decline associated with the drought was largely comparable across microhabitats, albeit perhaps somewhat larger in cacti. Finally, pre-drought bug crowding was higher in dwellings than in rocks or cacti and changed little during the drought–possibly with a downward trend in dwellings and an upward trend in cacti.

**Conclusions:**

*Triatoma brasiliensis* populations fared better in dwellings and rocks than in cacti during extreme drought. Estimates of microhabitat and drought effects on infestation, density, and crowding suggest that only a few cacti (*versus* many rocks and dwellings) represent good-quality habitat under such extremely harsh conditions. Our findings provide empirical support to the microhabitat-quality hierarchy hypothesis, and imply that *T*. *brasiliensis* can endure extreme climate by exploiting high-quality microhabitats, whether wild or man-made, in the semiarid Caatinga.

## Author summary

Triatomine bugs, the vectors of Chagas disease, feed on vertebrate blood and occupy diverse microhabitats in close association with their hosts. Each bug species is adapted to particular, ‘primary’ microhabitats. Many species, however, also use ‘secondary’ microhabitats, sometimes including man-made structures. Secondary wild microhabitats are probably of lower overall quality than primary microhabitats, whereas human dwellings can be of higher overall quality if they provide a stable and abundant blood supply. This suggests that there may be a ‘hierarchy’ of triatomine microhabitat quality–top-quality dwellings, high-quality primary microhabitats, and low-quality secondary wild microhabitats. To test this hypothesis, we measured the impact of a severe drought on *Triatoma brasiliensis* populations living in dwellings and wild habitats, both primary (rocks) and secondary (shrubby cacti). Dwelling and rock bug populations remained largely stable during the drought, whereas most cactus-living populations either became extinct or shrunk to an undetectable size. A few cacti, however, supported relatively large bug colonies even during the drought, suggesting that they were of higher-than-average quality. Our findings provide support to the microhabitat-quality hierarchy hypothesis, and show that *T*. *brasiliensis* (and perhaps other triatomines) can endure extreme climate by exploiting (natural or man-made) high-quality microhabitats.

## Introduction

*Trypanosoma cruzi*, the etiological agent of Chagas disease, is transmitted primarily by blood-sucking bugs known as triatomines [1]. Vector-borne transmission is restricted to the Americas, where recent estimates suggest that 4–5 million people carry *T*. *cruzi* [2]. American triatomine-bug species, of which over 130 are known, have adapted to highly diverse eco-regions ranging from rainforests to deserts; within them, the bugs associate with their vertebrate hosts in similarly diverse microhabitats–from underground burrows to tree-canopy epiphytes [1,3–7]. Table 1 summarizes a rough, yet broadly accepted, classification distinguishing triatomine species that are primarily terrestrial or primarily arboreal [3,5]. Some populations of a few species can also exploit man-made habitats, where they feed on the blood of people and domestic animals; these ‘synanthropic’ triatomines are the main vectors of human Chagas disease [1,3,6,7].

**Table 1:** Wild habitats and microhabitats of triatomine bugs, with a few examples.

Habitat	Microhabitat	Examples
Terrestrial	Underground (wildlife burrows, tree root cavities)	*Panstrongylus geniculatus*, *Triatoma protracta*
	Caves	*Cavernicola pilosa*, *T*. *mopan*, *Hermanlentia matsunoi*
	Among rocks/stones	*T*. brasiliensis*, Andean *T*. *infestans*, *T*. *costalimai*
	Ground bromeliads	*T*. *guasayana*, *P*. *howardi*
	Shrubby cacti	*T*. brasiliensis*
Arboreal	Hollow trees/tree holes	*P*. *megistus*, Chacoan *T*. *infestans*, *T*. *sordida*, *Eratyrus mucronatus*
	Under tree bark	*T*. *pseudomaculata*, *Belminus herreri*
	Epiphytic bromeliads	*T*. *tibiamaculata*, *Rhodnius domesticus*
	Palm crowns	Most *Rhodnius* spp. including *R*. *prolixus*
	Nests	*Psammolestes* spp., *T*. *delpontei*

*Triatoma brasiliensis, our study species, is primarily rock-dwelling but also occupies shrubby cacti

**Fig 1:**
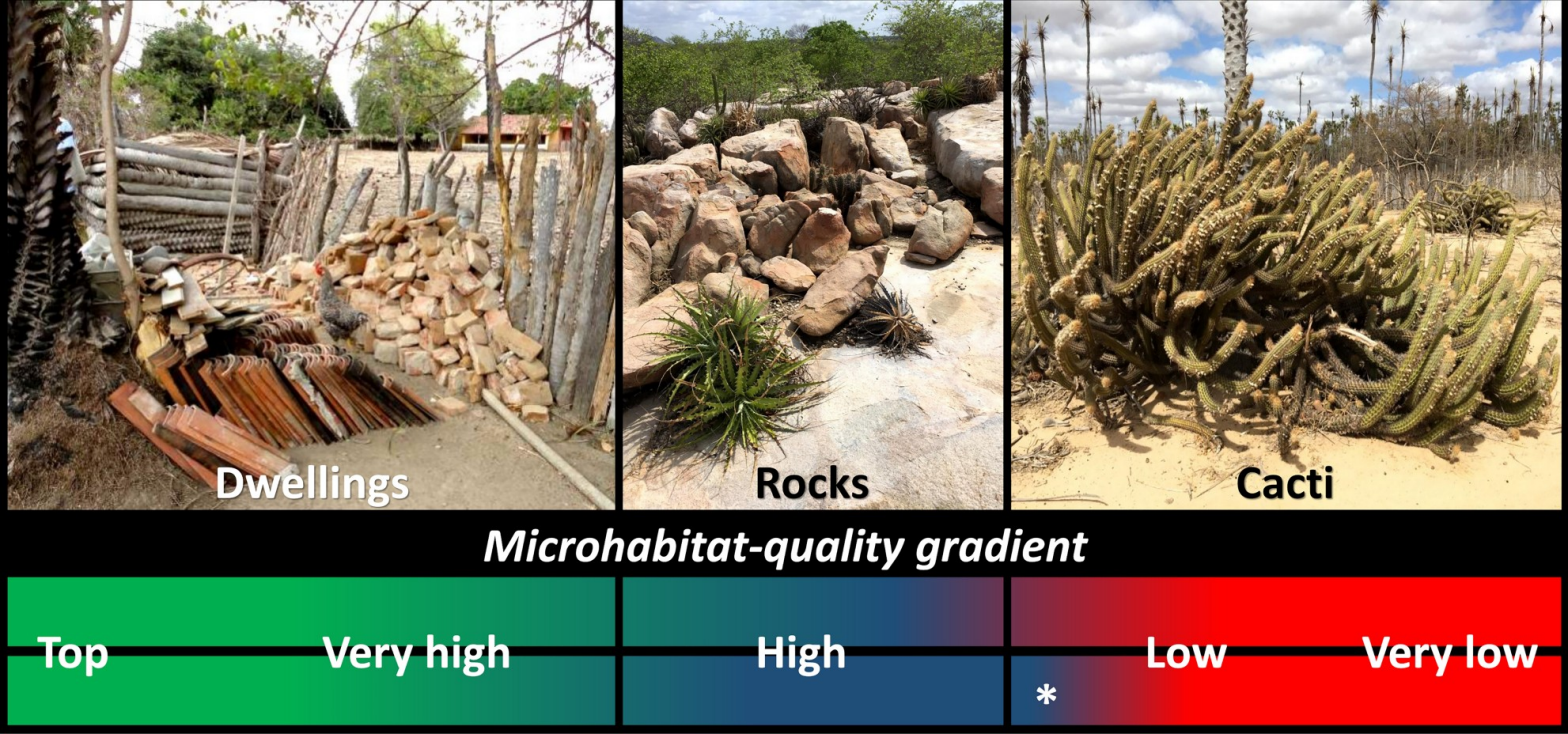
The microhabitats of *Triatoma brasiliensis*: Human dwellings, rocky outcrops, and shrubby cacti. The lower panel illustrates the hypothesis that microhabitat quality varies along a gradient running from top-quality dwellings through high-quality rock microhabitats (the primary habitat of the species) to lower-quality cacti (most likely secondary habitat); alternatively, a small minority of cacti may also represent high-quality habitat (white asterisk).

One distinctive feature of synanthropic triatomine populations is that they can build very high-density colonies [1,3,6–9]. Wild populations, in contrast, are almost always found in relatively small foci, with denser colonies reported only on occasion from particularly suitable microhabitats–e.g., those in which many hiding sites are available and resident vertebrates provide a stable food supply [6,7]. This suggests that there is variation in the quality of the microhabitats a triatomine-bug species can inhabit. Such variation can be viewed as defining a gradient that runs from the many zero-quality habitats that remain unoccupied (despite being available) to the few high-quality habitats that can sustain large breeding colonies [6–10] (Fig 1). This view may be framed as the ‘microhabitat-quality hierarchy’ hypothesis. In general terms, this hypothesis predicts that triatomine-bug populations occupying higher-quality microhabitats should fare overall better than those exploiting lower-quality microhabitats–and, importantly, that the difference should become particularly evident as environmental conditions get harsher [6,7,10–12]. Here, we use a unique dataset on wild and synanthropic *Triatoma brasiliensis* populations studied under both regular and exceptionally harsh climatic conditions to provide an empirical test of this hypothesis.

*Triatoma brasiliensis* is the most important vector of Chagas disease in the Caatinga of north-eastern Brazil [1,3,6,13]. The Caatinga is a semiarid scrubland/woodland eco-region where rainfall is scarce (~250–1500 mm/year) and highly irregular, with a dry season that can last up to 7–11 months in typical years [14]. Severe droughts, in addition, are common, with one- to five-year periods in which rainfall can drop by 50% [15]. *Triatoma brasiliensis* evolved in this harsh environment [3,4,16,17] and is well adapted to survive and reproduce in very dry/hot environments [17–20]. In particular, *T*. *brasiliensis* is common in rocky-outcrop habitats (Fig 1), where if feeds on wild rodents and other vertebrates [1,3,6,20–27]. In rock-free sedimentary lowlands, wild *T*. *brasiliensis* occupy *Pilosocereus* sp. shrubby cacti (Fig 1) in association with rodents. The species has never been reported from arboreal habitats in the wild [23,28–30]. Finally, *T*. *brasiliensis* often infest human dwellings (Fig 1) in rural areas where rock-outcrops, shrubby cacti, or both sustain wild populations that may act as the sources of dwelling-infesting and reinfesting bugs [1,3,6,13,21,23–27,31–35].

*Triatoma brasiliensis* belongs to the ‘*T*. *brasiliensis* complex’, a group of closely-related, saxicolous (i.e., rock-dwelling) species that almost certainly evolved in rocky habitats [1,3,4,16,17,21,29]. Rocky outcrops are therefore regarded as the primary habitat of *T*. *brasiliensis* [1,3] (Fig 1). Occupation of shrubby cacti, on the other hand, seems secondary–the originally rock-dwelling bugs likely followed their rodent hosts as they adapted to exploit cactus habitats in sedimentary lowlands [28,32]. Cacti, in addition, vary more in size and architectural complexity, and sustain overall smaller and less stable vertebrate populations, than rocky outcrops; this likely leads to sharper variation in the spatial-temporal availability of key resources (adequate shelter, vertebrate blood) for cactus-dwelling than for rock-dwelling bugs. Shrubby cacti, then, probably represent secondary, lower-quality habitat for *T*. *brasiliensis* [28,32] (Fig 1). Finally, the fact that *T*. *brasiliensis* is a notorious domestic pest, with frequent infestation of man-made structures and some high-density colonies [1,3,6,13,21,23–27,31–35], suggests that human dwellings, although not primary habitat, are high-quality microhabitats for the species (Fig 1). More generally, some human dwellings provide a particularly abundant and stable supply of shelter and food to synanthropic triatomines; in the absence of bug-control interventions, therefore, such dwellings are regarded as being of higher quality than wild microhabitats [1,3,6–9].

**Fig 2:**
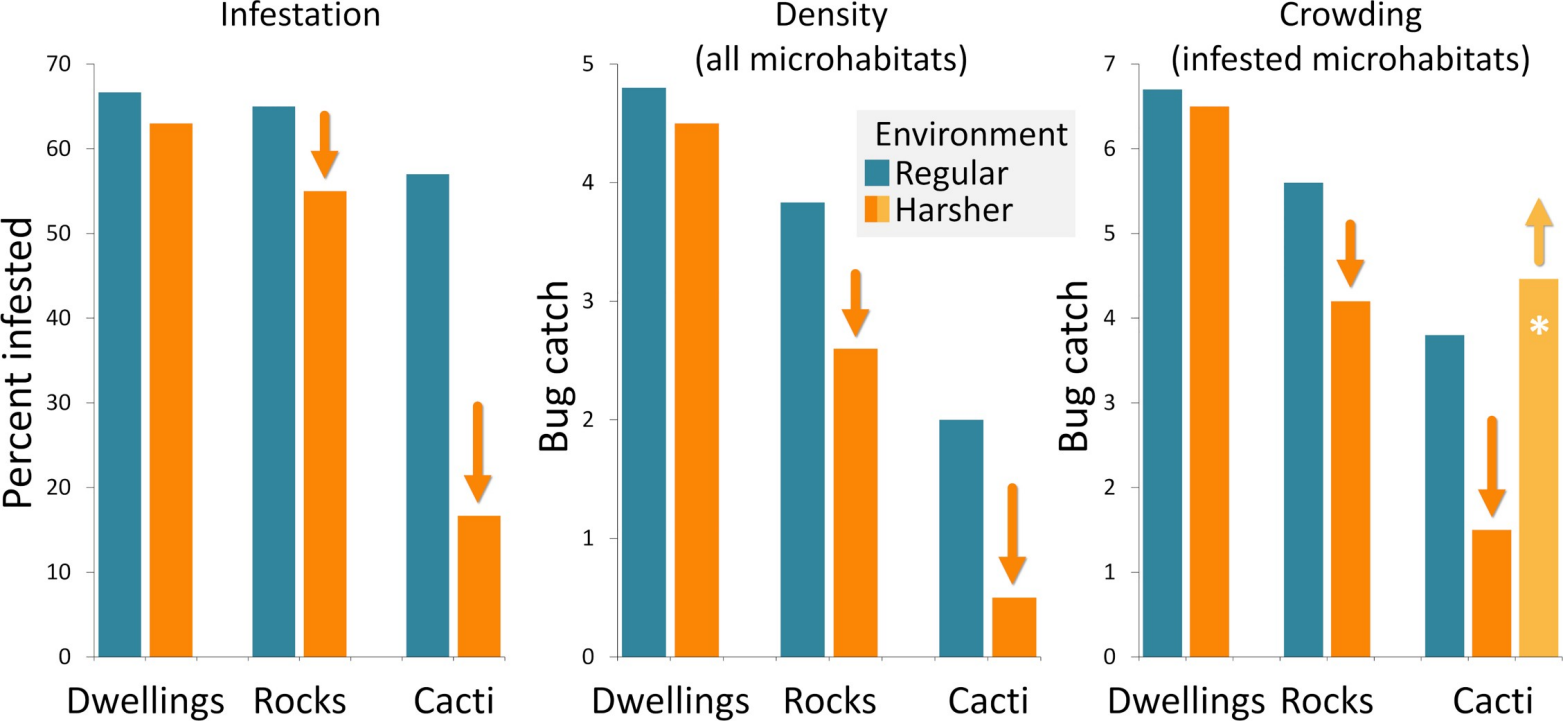
The microhabitat-quality hierarchy hypothesis: Predictions about habitat and drought effects. Arrows in the plots emphasize drought-associated changes predicted by the microhabitat-quality hierarchy hypothesis. Note that we illustrate two alternatives for bug crowding in cacti–either it declines with the drought, suggesting that virtually all cacti are low-quality for the bugs (darker orange), or it increases, suggesting that a few cacti are high-quality and can act as ‘safe havens’ that sustain relatively large colonies (lighter orange, with a white asterisk emphasizing the link with Fig 1).

The microhabitat-quality hierarchy hypothesis makes some key predictions in terms of (i) the proportion of microhabitats where bugs are found (or *infestation*); (ii) the number of bugs caught (per unit catch-effort) per microhabitat (or bug *density*); or (iii) the number of bugs caught (per unit catch-effort) per infested microhabitat (or bug *crowding*) (see ref. [6]). In particular, this hypothesis makes the following predictions (see Fig 2):

1. Under regular environmental conditions, infestation by *T*. *brasiliensis* should be similar across microhabitats, yet density and crowding should decline moderately from dwellings to rock microhabitats and from rock microhabitats to cacti;
2. Under harsher environmental conditions, infestation, density, and crowding should all remain largely stable (perhaps with a slight decline) in the highest-quality dwelling microhabitats, but should decline in wild habitats–moderately in the higher-quality rock microhabitats and more sharply in the lower-quality cactus microhabitats. If, however, enough resources remain available in a small minority of particularly suitable cacti (e.g., large, architecturally complex cacti that sustain stable wild-rodent populations), so that bug populations in those few cacti change little as environmental conditions get harsher, then density should decline more sharply than crowding in cactus microhabitats (Figs 1 and 2).

To investigate *T*. *brasiliensis* responses to harsher environmental conditions, we compared infestation, density, and crowding of bug populations sampled in human dwellings, rocky outcrops, and shrubby cacti before (or at the outset) and at the end of one of the most severe droughts on record in the Caatinga [15].

## Methods

### Study setting and timeline

Fieldwork took place in the municipalities of Russas (04°56’S, 37°58W) and Jaguaruana (04°50’S, 37°47’W), state of Ceará, Brazil. Both lie within the lower Jaguaribe river basin, whose rock-free sedimentary lowlands are bounded by crystalline-basement uplands in which rocky outcrops are widespread [22,28,32,33]. The original Caatinga scrubland/woodland vegetation is overall less disturbed in upland than in lowland areas, where only small preserved patches remain; *Copernicia prunifera* palms and *Pilosocereus* sp. shrubby cacti are common in those patches. Local rural dwellings usually comprise a substandard house with mud/clay or brick walls and timber-and-tile roofs plus a peridomestic area in which henhouses, corrals, pigsties, and piles of timber, tiles, and bricks are common [23,32,33,35] (Fig 1).

**Fig 3:**
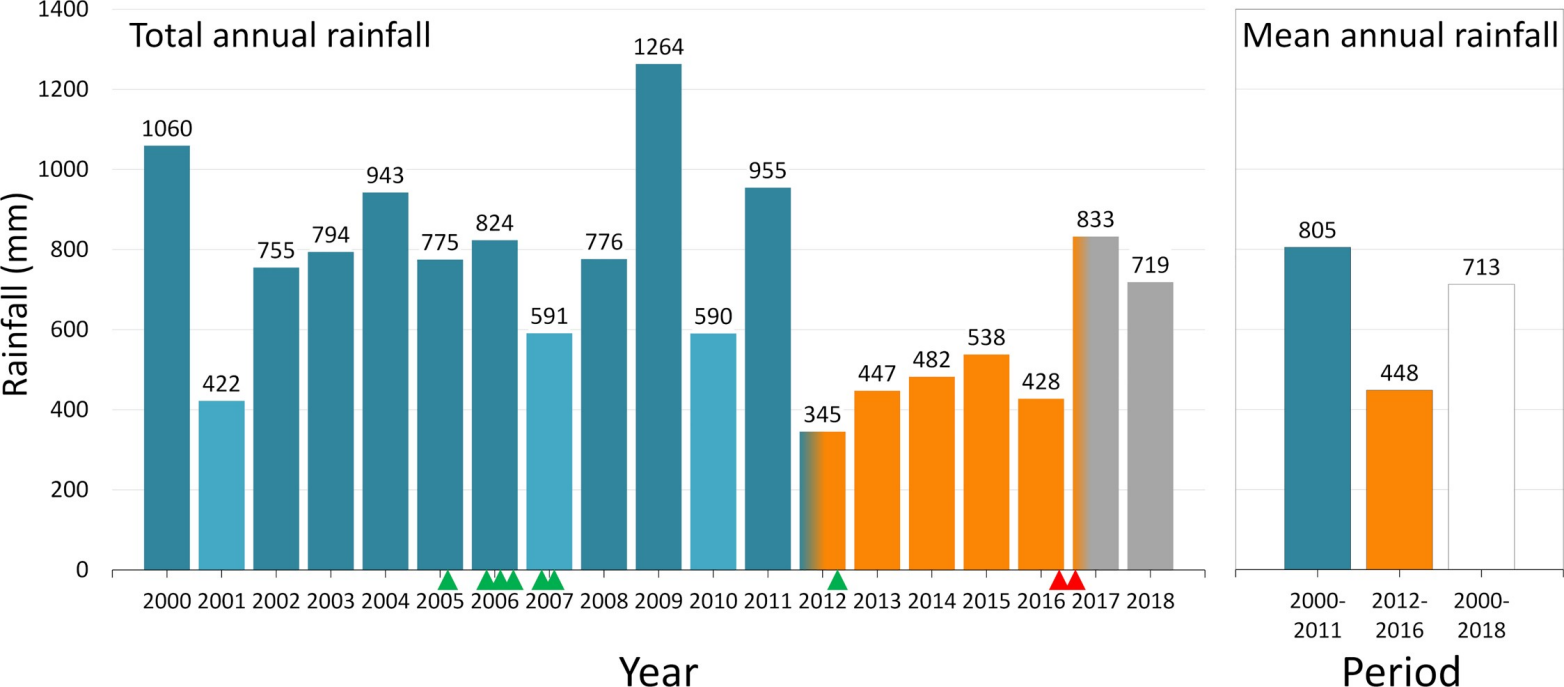
Rainfall in the study area, 2000–2018. Total annual and mean annual rainfall for the pre-drought (2000–2011, blue), drought (2012–2016, orange), and post-drought (2017–2018, grey) periods. Blue–orange gradation indicates that drought effects were barely perceptible in 2012, and orange–grey gradation that drought effects were still at its peak in early 2017. Lighter-blue bars indicate pre-drought years with rainfall below the overall mean (713 mm; empty bar in the right-hand panel). Pre-drought field trips are indicated by green arrowheads, and drought field trips by red arrowheads, along the *x*-axis. The graphs summarize daily rainfall data from the Russas rainfall station (Fundação Cearense de Meteorologia e Recursos Hídricos; data available at http://www.funceme.br/).

We sampled *T*. *brasiliensis* populations in December 2016 and January 2017, when the 2012–2016 severe drought hit its highest point (Fig 3). This sampling included 34 rural dwellings, 174 rock microhabitats, and 217 shrubby cacti (Table 2). During fieldwork, we noticed that some *T*. *brasiliensis* populations, and in particular those in wild microhabitats, were rarer and less dense than we would have expected based on our 10-year experience of field research in the region [22,23,32–35]. This led us to hypothesize a strong negative impact of the drought on *T*. *brasiliensis* populations occupying the lower-quality wild microhabitats–and an overall milder impact on populations occupying the higher-quality man-made microhabitats (Figs 1 and 2). Testing this hypothesis required comparing *T*. *brasiliensis* populations sampled in different microhabitats and under regular and severe climatic conditions (Fig 2). To do this, we used ‘pre-drought’ field data collected by our group in the same study area, yet either before the 2012–2016 drought [22] or right after its onset [28,32,34] (Fig 3). These pre-drought data cover six field trips conducted in 2005–2007 to sample rock microhabitats [22] plus dwellings and shrubby cacti studied in 2012 [28,32,34], when the impact of the drought was not yet perceptible (Fig 3). Overall, we compiled data from 606 *T*. *brasiliensis* populations– 66 sampled in human dwellings, 279 in rock microhabitats, and 261 in *Pilosocereus* sp. shrubby cacti (Table 2). We note, however, that some of these populations were sampled at different time-points in the same microhabitats (e.g., [22]); in addition, microhabitats sampled at any given occasion tended to occur in spatial clusters. Even if repeated sampling of individual microhabitats was separated by at least three months and the microhabitats we sampled were located at least ~100 m from each other, these data cannot be treated as independent. We account for these sources of non-independence via random effects in generalized linear mixed models (see below). Triatomine-bug sampling was done with two methods–direct manual searches and live-baited sticky traps [22,23,32–36]. We controlled for unequal bug-catch effort among individual microhabitats using an offset variable in our models (see below); to derive this variable, we considered one person-hour of manual searching (defined as one hour of bug searching/catching by one person) as roughly equivalent to one live-baited trap-night (defined as one trap operated over one night). We also note that we defined our ‘rock-microhabitat sampling unit’ as a discrete site that could potentially provide shelter to a *T*. *brasiliensis* colony; each ‘unit’ was usually a crevice or a space beneath a rock or a cluster of rocks (such as that shown in Fig 1). See S1 Dataset for the raw data.

**Table 2:** Descriptive and exploratory analyses: Microhabitat infestation by *Triatoma brasiliensis*, bug density, and bug crowding.

	Human dwellings			Rocks			Shrubby cacti			All microhabitats		
	Pre-drought	Drought	Total	Pre-drought	Drought	Total	Pre-drought	Drought	Total	Pre-drought	Drought	Total
Sampled	32	34	66	105	174	279	44	217	261	181	425	606
Infested	16	12	28	65	118	183	21	8	29	102	138	240
Infestation	0.50	0.35	0.42	0.62	0.68	0.66	0.48	0.04	0.11	0.56	0.33	0.40
85% CI	0.38–0.62	0.35–0.48	0.34–0.51	0.55–0.68	0.63–0.73	0.61–0.70	0.37–0.58	0.02–0.06	0.09–0.14	0.51–0.62	0.29–0.36	0.37–0.43
Bug catch	520	320	840	702	568	1270	157	49	206	1379	937	2316
Density (CPUE)*	8.13	2.50	5.23	1.67	1.17	1.36	0.55	0.05	0.14	2.54	0.70	1.25
85% CI	3.87–12.38	0.57–4.43	2.93–7.53	1.24–2.10	0.99–1.34	1.16–1.55	0.31–0.79	0.02–0.09	0.09–0.19	1.72–3.36	0.53–0.88	0.98–1.53
Crowding (CPUE)*	16.25	7.08	12.32	2.70	1.72	2.07	1.15	1.44	1.23	4.51	2.17	3.16
85% CI	8.58–23.92	1.79–12.37	7.42–17.22	2.07–3.28	1.49–1.95	1.80–2.34	0.71–1.59	0.76–2.12	0.87–1.58	3.11–5.91	1.67–2.66	2.50–3.83

CI, confidence interval

*Frequency histograms of bug catch per unit effort (CPUE) in each microhabitat and period are presented in S1 Fig

### Data and data analyses

Our data are of two main types. First, we distinguished microhabitats in which at least one *T*. *brasiliensis* was found (coded 1) from those in which none was (coded 0); this binomial variable allowed us to test the predictions about infestation probabilities made by the microhabitat-quality hierarchy hypothesis (Fig 2). Second, we recorded the number of bugs caught in each microhabitat, and used this count variable to test predictions about changes in bug density and crowding (Fig 2). We built dummy indicator variables for microhabitat type (dwellings, rock microhabitats, or cacti) and for severe drought (coded 1 for microhabitats sampled in 2016–2017 and 0 otherwise).

We used R 3.5.1 [37] for all our analyses. Descriptive and exploratory analyses included data tabulation/graphing and the calculation of the proportions of infested microhabitats, the mean number of bugs caught per unit effort in all microhabitats (i.e., density), and the mean number of bugs caught per unit effort in microhabitats where at least one bug had been found (i.e., crowding). We preliminarily compared these metrics across habitat types and between periods by calculating and plotting score confidence intervals (CIs) around proportions [38] and *t*-test-based CIs around means [39]. We did these analyses using the *stats* 3.5.1 and *Hmisc* 4.1–1 R packages [37,40].

We aimed at formally testing the predictions of the microhabitat-quality hierarchy hypothesis while taking into account unequal sampling effort and the non-independence of spatially-clustered observations. To do this, we fitted a series of generalized linear mixed models (GLMMs) and evaluated them using second-order Akaike’s information criterion scores (AICc) [41,42]. We modeled infestation with binomial GLMMs; AICc selected the complementary log-log (cloglog) link-function instead of the canonical logit link. Preliminary analyses also showed that the negative binomial distribution fits our bug-count data better than the Poisson; because crowding data contain no zeros, we modeled this variable using a zero-truncated negative binomial distribution [43]. All our models included (i) the log-transformed bug-catch effort as an offset, which made bug detection/catch data comparable across microhabitats, and (ii) a 10-cluster random effect (coded ‘C_1’ to ‘C_10’ in S1 Dataset) to account for the likely non-independence of temporally-replicated or spatially-clustered observations. The models fall in the following categories (see Table 3):

- *Null models*, representing the hypothesis that neither microhabitat type nor drought have any effects on infestation, density, or crowding;
- *Microhabitat models*, representing the hypothesis that infestation, density, or crowding may vary between microhabitats, but without measurable drought effects;
- *Drought models*, representing the hypothesis that the drought, but not microhabitat type, has measurable effects on infestation, density, or crowding;
- *Joint microhabitat/drought models*, including (a) *additive models* representing the hypothesis that drought effects are consistent across microhabitats and microhabitat effects are similar in pre-drought and drought periods, and (b) *interaction models* representing the hypothesis that drought effects differ among microhabitats and habitat effects may vary with the drought.

**Table 3:** Microhabitat and drought effects on *Triatoma brasiliensis*: Models and hypotheses.

Model types	Model structure* [generic model code]	Specific hypothesis
Null	*Y*~offset(log-effort)+random(cluster) [m0]	No habitat or drought effects: outcome *Y* varies randomly across microhabitat types; no variation in pre-drought *vs*. drought periods (*accounting for unequal bug-catch effort and cluster-level non-independence*)*
Microhabitat	*Y*~microhabitat type	Outcome *Y* varies across different microhabitat types, but there are no drought effects*
	*Y*~rock+cactus [m1]	Outcome *Y* is different in each of the three microhabitat types, but there are no drought effects*
	*Y*~dwelling [m2]	Outcome *Y* differs between man-made and wild microhabitats, but there are no drought effects*
	*Y*~rock [m3]	Outcome *Y* differs between primary (rocks) and secondary (dwellings and cacti) microhabitats, but there are no drought effects*
	*Y*~cactus [m4]	Outcome *Y* differs between higher-quality (dwellings and rocks) and lower-quality (cacti) microhabitats, but there are no drought effects*
Drought	*Y*~drought [m5]	Only the drought makes a difference; outcome *Y* does not vary across microhabitat types*
Joint-additive	*Y*~microhabitat+drought	Drought effects are the same irrespective of microhabitat type, and habitat effects are the same irrespective of drought*
	*Y*~rock+cactus+drought [m6]	Outcome *Y* differs among all three microhabitat types, and also changes (independently of microhabitat type) with the drought*
	*Y*~dwelling+drought [m7]	Outcome *Y* differs between man-made and wild microhabitats, and also changes (independently of microhabitat) with the drought*
	*Y*~rock+drought [m8]	Outcome *Y* differs between primary and secondary microhabitats, and also changes (independently of microhabitat) with the drought*
	*Y*~cactus+drought [m9]	Outcome *Y* differs between higher- and lower-quality microhabitats, and also changes (independently of microhabitat) with the drought*
Joint-interactions	*Y*~microhabitat×drought	Drought effects differ among microhabitats, and habitat effects depend on whether or not there is drought*
	*Y*~rock×drought+cactus×drought [m10]	Drought effects are different in each of the different microhabitat types*
	*Y*~dwelling×drought [m11]	Drought effects are different in man-made *vs*. wild (whether rocks or cacti) microhabitats*
	*Y*~rock×drought [m12]	Drought effects are different in primary (rocks) *vs*. secondary (dwellings, cacti) microhabitats*
	*Y*~cactus×drought [m13]	Drought effects are different in higher-quality (dwellings and rocks) *vs*. lower-quality (cacti) microhabitats*

*All models include a sampling-effort offset and a cluster random effect (shown only in ‘m0’), and therefore account for unequal bug-catch effort and cluster-level non-independence; *Y* represents the outcome (infestation, bug density, or bug crowding) being modeled

For example, a rather sophisticated view of the microhabitat-quality hierarchy hypothesis is represented by interaction microhabitat × drought models stating, e.g., (i) that the drought should not have measurable effects on infestation, density, or crowding in human dwellings; (ii) that infestation, density, and crowding should decline moderately in rock microhabitats with the drought; and (iii) that infestation and density should decline moderately in cacti–where crowding, however, may even increase with the drought (Fig 2, Table 3). This can be represented by the ‘full’ model

Y∼rock×drought+cactus×drought+offset(log‐effort)+random(cluster)+ε,

in which *Y* represents the response variable (infestation, density or crowding), × represents an interaction between microhabitat type and drought, the offset adjusts for unequal bug-catch effort, the random-intercepts term accounts for cluster-level non-independence of observations, and ε is residual error. An alternative model could represent the competing specific hypothesis that drought effects should only be apparent in the lower-quality cactus microhabitats, with the drought having negligible effects on populations occupying high-quality dwelling or rock microhabitats (Table 3):

Y∼cactus×drought+offset(log‐effort)+random(cluster)+ε.

Our models, including model type, general model structure, and a brief outline of the specific hypothesis each represents, are summarized in Table 3. We fitted and evaluated infestation and density models using *lme4* 1.1–19 [44], and crowding models using *glmmADMB* 0.8.3.3 [45]. For a given dataset (infestation, density, or crowding), models with lower AICc scores have more support from the data; as a rough guidance, models with AICc scores ~4–6 units larger than the lowest-score model have little to no support from the data [41,42]. We also used AICc model weights (*w*_*i*_) as a measure of the evidence in favor of each model [41]. We finally quantitatively evaluated, for each outcome variable, microhabitat and drought effects (and their interactions) as estimated (i) by the most competitive models (AICc < 2.0 units from the top-ranking model’s AICc) in each model set and (ii) across all models in each set by computing model-averaged coefficients and unconditional CIs [41] in the package *MuMIn* 1.42.1 [46]. We note that, for consistency with our AIC-based approach (see ref. [47]), we present all point estimates with 85% CIs.

### Ethics statement

This study is part of a research program on Chagas disease ecoepidemiology reviewed and approved by the Fiocruz Research Ethics Committee (CEP/Fiocruz 139/01), by the Fiocruz Committee on Animal Research (CEUA/Fiocruz L-026/2017), and by the Brazilian Environmental Agency (IBAMA/Sisbio 14323–6). All household heads provided informed consent prior to dwelling inspections.

## Results

### Descriptive and exploratory analyses

**Fig 4:**
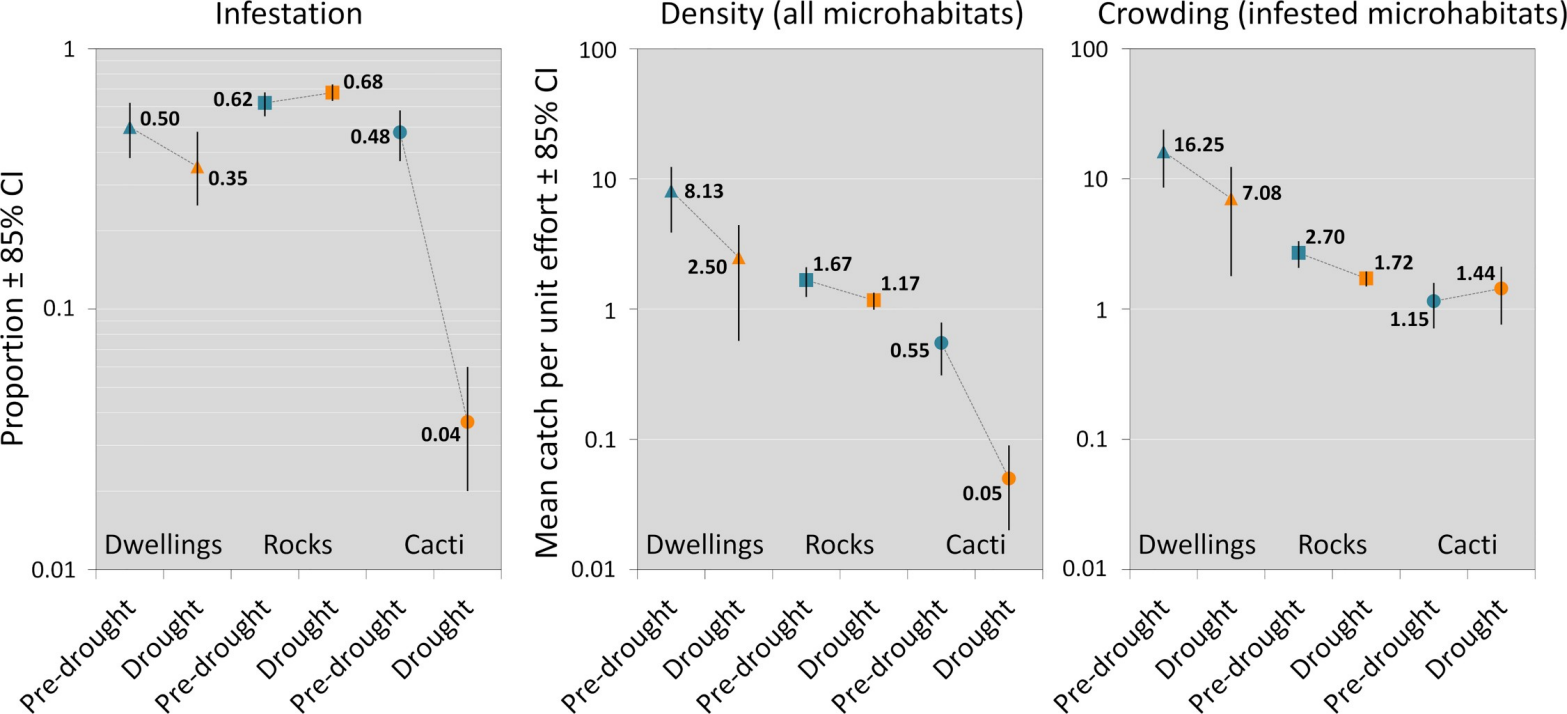
Observed microhabitat infestation by *Triatoma brasiliensis*, bug density, and bug crowding. Summary metrics by microhabitat (dwellings = triangles; rocks = squares; cacti = circles) both before (‘pre-drought’, blue) and during the drought (‘drought’, orange), with 85% confidence intervals (CI). Note that *y*-axes are on log10 scale.

Table 2 presents a summary of our observations and exploratory analyses; the main results are graphically shown in Fig 4. Pre-drought infestation was similarly high (~50–60%) across microhabitat types. Severe drought was associated with a slight decrease of dwelling infestation and with no measurable change in rock infestation; in shrubby cacti, in contrast, infestation fell by one order of magnitude–from nearly 50% to about 4% (Table 2, Fig 4). Pre-drought bug density and crowding decreased from dwellings to rocks to cacti. Density then decreased across all microhabitat types during the drought; it did so moderately in dwellings and rocks, yet very sharply (again by an order of magnitude) in cacti (Table 2, Fig 4). Finally, bug crowding also decreased moderately in dwellings and rocks, paralleling changes in density, but increased, even if slightly, in cacti–from 1.15 to 1.44 bugs per unit catch-effort per infested cactus (Table 2, Fig 4).

### Generalized linear mixed models–infestation

**Fig 5:**
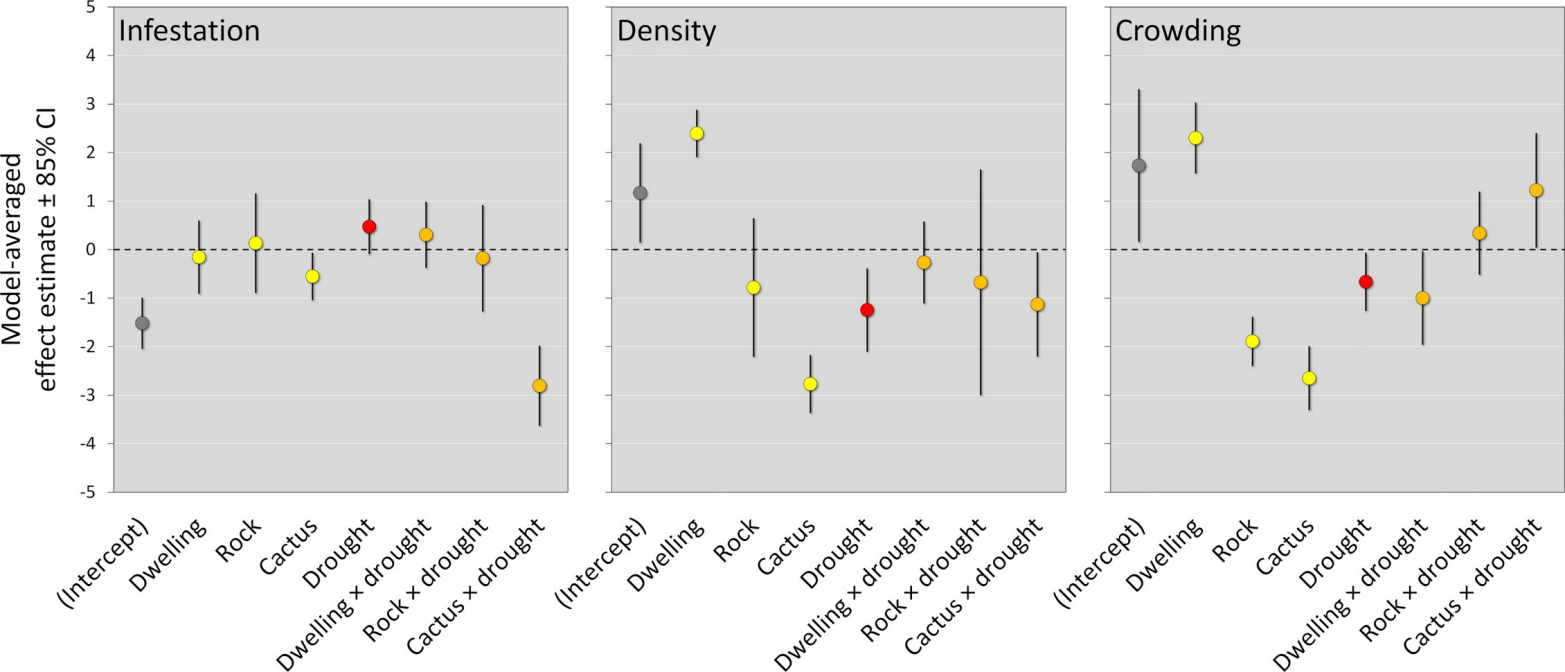
Microhabitat and drought effects on wild and synanthropic *Triatoma brasiliensis* populations: Model-averaged slope-coefficient estimates and confidence intervals (CI). Microhabitat effects are represented in yellow, drought effects in red, and microhabitat × drought interactions in orange; intercepts (grey) are included for reference; the dashed horizontal line at zero indicates no effect.

We fitted 14 binomial GLMMs to evaluate the relative support for each of our specific infestation hypothesis (see Table 3). Table 4 shows that the better-performing models were joint interaction models; simpler microhabitat-only, drought-only, or joint additive models had essentially no support from the data. The top-ranking model (*w*_*i*_ = 0.882) had much more support than the second-ranking model (ΔAICc > 4; Table 4). We therefore found strong evidence [41,42] in favor of the specific hypothesis stating that the effects of the drought on infestation were different in higher-quality (dwellings and rocks) *vs*. lower-quality (cacti) microhabitats–relative to the competing hypotheses in Table 3 and after accounting for unequal sampling effort and cluster-level non-independence of observations. The numerical output of the top-ranking model is presented in Table 5, and model-averaged coefficients for all variables in Fig 5. These quantitative results suggest (i) that microhabitat effects were relatively small, with pre-drought infestation probably slightly less frequent in cactus than in dwellings or rocks, and (ii) that the drought had a strong negative effect on cactus infestation–but only a small impact, effectively indistinguishable from zero, on dwelling and rock infestation (Table 5, Fig 5).

**Table 4:** Infestation models: Structure and performance. The only competitive model (ΔAICc < 2.0) is in bold typeface.

Model type	Model structure*	Code	*k*	AICc	ΔAICc	*w*_*i*_	log-Likelihood
**Joint-interaction**	***Y*~cactus×drought**	**im13**	**5**	**555.38**	**0**	**0.882**	**−272.641**
Joint-interaction	*Y*~rock×drought+cactus×drought	im10	7	559.41	4.03	0.118	−272.613
Joint-additive	*Y*~cactus+drought	im9	4	575.32	19.94	0.000	−283.627
Joint-additive	*Y*~rock+cactus+drought	im6	5	577.04	21.66	0.000	−283.469
Joint-additive	*Y*~dwelling+drought	im7	4	581.62	26.24	0.000	−286.777
Joint-interaction	*Y*~dwelling×drought	im11	5	583.24	27.86	0.000	−286.571
Drought	*Y*~drought	im5	3	585.37	29.99	0.000	−289.667
Microhabitat	*Y*~cactus	im4	3	586.35	30.97	0.000	−290.156
Joint-additive	*Y*~rock+drought	im8	4	587.33	31.95	0.000	−289.631
Microhabitat	*Y*~rock+cactus	im1	4	588.19	32.80	0.000	−290.059
Joint-interaction	*Y*~rock×drought	im12	5	589.33	33.95	0.000	−289.616
Microhabitat	*Y*~dwelling	im2	3	605.48	50.10	0.000	−299.721
Null	*Y*~offset(log-effort)+random(cluster)*	im0	2	621.16	65.77	0.000	−308.568
Microhabitat	*Y*~rock	im3	3	623.01	67.62	0.000	−308.482

*All models include a sampling-effort offset and a cluster random effect (shown only in the ‘null’ model, ‘im0’), and therefore account for unequal bug-catch effort and cluster-level non-independence; *Y* represents the outcome being modeled (here, infestation); *k*, number of parameters; AICc, second-order Akaike’s information criterion; ΔAICc, difference of AICc scores between each model and the lowest-AICc (top-ranking) model; *w*_*i*_, model Akaike weight

**Table 5:** Top-ranking infestation model (im13; *w*_*i*_ = 0.882): Structure and numerical estimates.

Effects	Term	Estimate	SE	85% CI	
Fixed	Intercept	−1.511	0.353	−2.124	−1.059
	Cactus	−0.556	0.338	−1.043	−0.062
	Drought	0.464	0.381	−0.082	1.049
	Cactus × drought	−2.799	0.567	−3.649	−1.997
Random	Cluster (variance)	0.272	-	0.213	1.028

*w*_*i*_, model Akaike weight; SE, standard error; CI, profile-likelihood confidence interval

### Generalized linear mixed models–density

Bug-density negative binomial models, ranked by AICc, are presented in Table 6. Only two joint models (one additive, one with interactions) were competitive (ΔAICc < 2.0; Σ*w*_*i*_ = 0.892); their numerical output is presented in Table 7. These analyses suggest that bug density varied across microhabitat types and was also affected by the drought, with perhaps some moderate variation of drought effects across different microhabitats; as with infestation, microhabitat-only and drought-only models had little support from the data (Table 6). Model-averaged effect estimates are shown in Fig 5. These quantitative results suggest (i) that there were strong microhabitat effects, with pre-drought bug density declining from dwellings to rocks to cacti, and (ii) that the drought had moderate negative effects on bug density–with probably a somewhat stronger effect on cactus populations than in rock or dwelling populations (Table 7, Fig 5).

**Table 6:** Bug density models: Structure and performance. Competitive models (ΔAICc < 2.0) are in bold typeface.

Model type	Model structure*	Code	*k*	AICc	ΔAICc	*w*_*i*_	log-Likelihood
**Joint-additive**	***Y*~rock+cactus+drought**	**dm6**	**6**	**1994.43**	**0**	**0.614**	**−991.147**
**Joint-interaction**	***Y*~rock×drought+cactus×drought**	**dm10**	**8**	**1996.02**	**1.58**	**0.278**	**−989.887**
Joint-additive	*Y*~cactus+drought	dm9	5	1999.43	4.99	0.051	−994.665
Joint-interaction	*Y*~cactus×drought	dm13	6	1999.58	5.15	0.047	−993.721
Joint-additive	*Y*~dwelling+drought	dm7	5	2003.28	8.85	0.007	−996.591
Joint-interaction	*Y*~dwelling×drought	dm11	6	2005.12	10.69	0.003	−996.490
Microhabitat	*Y*~rock+cactus	dm1	5	2019.06	24.63	0.000	−1004.481
Microhabitat	*Y*~cactus	dm4	4	2024.57	30.14	0.000	−1008.254
Microhabitat	*Y*~dwelling	dm2	4	2031.97	37.53	0.000	−1011.951
Joint-additive	*Y*~rock+drought	dm8	5	2070.05	75.62	0.000	−1029.976
Joint-interaction	*Y*~rock×drought	dm12	6	2072.05	77.61	0.000	−1029.954
Drought	*Y*~drought	dm5	4	2073.90	79.47	0.000	−1032.917
Microhabitat	*Y*~rock	dm3	4	2139.64	145.20	0.000	−1065.784
Null	*Y*~offset(log-effort)+random(cluster)*	dm0	3	2142.21	147.78	0.000	−1068.085

*All models include a sampling-effort offset and a cluster random effect (shown only in the ‘null’ model, ‘dm0’), and therefore account for unequal bug-catch effort and cluster-level non-independence; *Y* represents the outcome being modeled (here, bug density); *k*, number of parameters; AICc, second-order Akaike’s information criterion; ΔAICc, difference of AICc scores between each model and the lowest-AICc (top-ranking) model; *w*_*i*_, model Akaike weight

**Table 7:** Competitive density models (ΔAICc < 2.0; ∑*w*_*i*_ = 0.892): Structure and numerical estimates.

Model code	*w*_*i*_	Effects	Term	Estimate	SE	85% CI	
dm6	0.614	Fixed	Intercept	1.397	0.609	0.401	2.281
			Rock	−0.976	0.406	−1.607	−0.428
			Cactus	−2.910	0.337	−3.406	−2.432
			Drought	−1.520	0.298	−1.955	−1.097
		Random	Cluster (variance)	2.358	-	0.939	2.599
dm10	0.278	Fixed	Intercept	0.864	0.700	−0.249	1.867
			Rock	−0.351	1.588	−2.787	2.232
			Cactus	−2.490	0.443	−3.107	−1.883
			Drought	−0.638	0.653	−1.555	0.342
			Rock × drought	−0.675	1.615	−3.350	1.761
			Cactus × drought	−1.162	0.750	−2.278	−0.104
		Random	Cluster (variance)	2.009	-	0.864	2.405

*w*_*i*_, model Akaike weight; SE, standard error; CI, profile-likelihood confidence interval

### Generalized linear mixed models–crowding

Table 8 shows that there was more model-selection uncertainty, as measured by the slower rate of decline of *w*_*i*_ values, in crowding than in infestation or density models (see Tables 4 and 6). The two top-ranking models (ΔAICc < 2.0; Σ*w*_*i*_ = 0.565) were the same joint models that AICc selected as the best-performing density models (Tables 6 and 8). This suggests that, as for density, bug crowding varied across microhabitat types and was also affected by the drought–with this latter effect likely differing to some extent among different microhabitats. The third-ranking crowding model (‘cm2’, ΔAICc = 2.34), however, was a habitat-only model distinguishing man-made from wild microhabitats, and the fifth-ranking model (‘cm1’, ΔAICc = 2.59) was another habitat model distinguishing all microhabitat types (Table 8), thus providing some evidence that microhabitat effects, and in particular dwelling effects, might be stronger for bug crowding than for density. The numerical output of the competitive crowding models (Table 9) and model-averaged estimates (Fig 5) substantiate these views, showing (i) that pre-drought microhabitat effects were strong, with a sharp difference between the less crowded wild microhabitats and the much more crowded dwellings, and (ii) that drought effects were overall small and probably different across microhabitat types–somewhat negative in dwellings, close to zero in rocks, and somewhat positive in cacti (Table 9, Fig 5).

**Table 8:** Bug crowding models: Structure and performance. Competitive models (ΔAICc < 2.0) are in bold typeface.

Model type	Model structure*	Code	*k*	AICc	ΔAICc	*w*_*i*_	log-Likelihood
**Joint-additive**	***Y*~rock+cactus+drought**	**cm6**	**6**	**1368.28**	**0**	**0.394**	**-677.958**
**Joint-interaction**	***Y*~rock×drought+cactus×drought**	**cm10**	**8**	**1369.94**	**1.66**	**0.172**	**-676.658**
Microhabitat	*Y*~dwelling	cm2	4	1370.61	2.34	0.122	-681.222
Joint-additive	*Y*~dwelling+drought	cm7	5	1370.75	2.48	0.114	-680.248
Microhabitat	*Y*~rock+cactus	cm1	5	1370.87	2.59	0.108	-680.307
Joint-interaction	*Y*~dwelling×drought	cm11	6	1371.22	2.95	0.090	-679.432
Joint-interaction	*Y*~cactus×drought	cm13	6	1393.62	25.34	0.000	-690.628
Joint-additive	*Y*~rock+drought	cm8	5	1395.26	26.99	0.000	-692.503
Joint-additive	*Y*~cactus+drought	cm9	5	1396.07	27.79	0.000	-692.906
Microhabitat	*Y*~cactus	cm4	4	1396.29	28.01	0.000	-694.059
Joint-interaction	*Y*~rock×drought	cm12	6	1397.36	29.09	0.000	-692.501
Microhabitat	*Y*~rock	cm3	4	1400.04	31.77	0.000	-695.936
Drought	*Y*~drought	cm5	4	1411.48	43.20	0.000	-701.653
Null	*Y*~offset(log-effort)+random(cluster)*	cm0	3	1417.70	49.42	0.000	−1068.085

*All models include a sampling-effort offset and a cluster random effect (shown only in the ‘null’ model, ‘cm0’), and therefore account for unequal bug-catch effort and cluster-level non-independence; *Y* represents the outcome being modeled (here, bug crowding); *k*, number of parameters; AICc, second-order Akaike’s information criterion; ΔAICc, difference of AICc scores between each model and the lowest-AICc (top-ranking) model; *w*_*i*_, model Akaike weight

**Table 9:** Competitive crowding models (ΔAICc < 2.0; ∑*w*_*i*_ = 0.566): Structure and numerical estimates.

Model code	*w*_*i*_	Effects	Term	Estimate	SE	85% CI	
cm6	0.394	Fixed	Intercept	2.438	0.299	2.008	2.868
			Rock	−1.825	0.302	−2.260	−1.390
			Cactus	−2.517	0.390	−3.079	−1.956
			Drought	−0.635	0.197	−0.919	−0.352
		Random	Cluster (variance)	0.000	-	-*	-*
cm10	0.172	Fixed	Intercept	2.606	0.374	2.068	3.145
			Rock	−1.942	0.408	−2.530	−1.354
			Cactus	−2.957	0.485	−3.655	−2.258
			Drought	−1.035	0.552	−1.829	−0.240
			Rock × drought	0.340	0.595	−0.516	1.196
			Cactus × drought	1.223	0.818	0.046	2.401
		Random	Cluster (variance)	0.000	-	-*	-*

*w*_*i*_, model Akaike weight; SE, standard error; CI, profile-likelihood confidence interval

*CIs are not available for random-effects variance estimates from zero-truncated negative-binomial models in the version (1.42.1) of the R package *MuMIn* we used [46]; note, however, that cluster variance estimates were approximately zero, suggesting that model covariates explained most of the variation in crowding–so that there was virtually no among-cluster variance left to be explained by the random intercepts

## Discussion

In this study we have examined the notion that some of the habitats that Chagas disease vectors can occupy are of higher quality than others–i.e., that there is a ‘hierarchy’ of triatomine-bug microhabitat quality (Fig 1). We confronted some of the key predictions of this hypothesis with extensive field data gathered before and during an extreme climate event–the severe drought that ravaged the Caatinga in 2012–2016. Using *T*. *brasiliensis* as a case-study, we found compelling evidence for the often-assumed, yet seldom tested, idea that substandard rural dwellings are of higher quality for the bugs than the species’ wild microhabitats. Further, our findings indicate that rock microhabitats, where the primarily saxicolous *T*. *brasiliensis* probably evolved, are of higher quality than shrubby cacti–which most likely represent secondary habitat. However, our results also suggest that, even if cacti are of low average quality, a few high-quality cacti can act as ‘safe havens’ where relatively large *T*. *brasiliensis* populations survive through periods of extremely harsh environmental conditions; the same appears to be true, in contrast, of most rock and dwelling microhabitats (Figs 1 and 2).

Triatomines probably evolved from nest-dwelling, arthropod-feeding ancestors [1,4,48], and now occupy protected microhabitats in close association with their vertebrate hosts [1,3,5,7] (Table 1). The quality of those microhabitats is thought to depend on (i) whether the physical substrate is adequate for the bugs’ hiding, resting, foraging, mating, and egg-laying requirements; (ii) whether the microclimate is adequate for bug development, survival, and reproduction; and (iii) whether the vertebrate-blood supply is adequate in terms of quality, amount, and temporal stability [1,3,7,49]. Here, ‘adequate’ is defined relative to the physical substrate, microclimate, and blood sources each bug species is adapted to–i.e., relative to the characteristics of the ‘primary’ microhabitats where the species evolved. Many triatomine species, however, can also breed in ‘secondary’ microhabitats–including, in some cases, man-made habitats. Theory predicts that, all else being equal, secondary habitat should be of lower overall quality than the primary habitat each species is adapted to [50]. If, however, adequate substrate, microclimate, and blood supply are available in some secondary microhabitats, then they might become high-quality–possibly even higher-quality than the species’ primary habitat if, for example, food availability is enhanced or if there is ‘ecological release’ from predators or competitors [51,52]. In the absence of bug-control interventions, this appears to be the case of some human dwellings [1,3]. Further, the highly aggregated distribution of wild triatomine populations, with many small and very few large colonies [7], suggests that natural microhabitats, either primary or secondary, may also vary in their quality. While these observations clearly hint at the existence of a ‘hierarchy’ of triatomine-bug microhabitat quality, from top-quality dwellings through good-quality primary to poor-quality secondary wild habitats (Fig 1), a formal test of this hypothesis was apparently unavailable before this report.

Our results provide empirical support for the view that human dwellings can be higher-quality habitat for *T*. *brasiliensis* than the species’ natural habitats, whether primary (rock microhabitats) or secondary (shrubby cacti) (Figs 1 and 2). First, pre-drought infestation with *T*. *brasiliensis* was about as frequent in dwellings as it was in rocks or cacti, yet bug density and crowding were clearly higher in dwellings (Table 2, Figs 4 and 5). This suggests that, in regular-climate periods (and in the absence of systematic vector control), the bugs use dwellings about as often as they use their natural habitats, yet synanthropic colonies grow larger, on average, than wild ones. Second, dwelling infestation varied little, perhaps with a slight decline, as environmental conditions got extremely harsh; this was similar to what was seen in the bugs’ primary rocky habitat, but not in secondary cactus habitats, where infestation dropped by about 10-fold (Table 2, Figs 4 and 5). Dwelling bug density and crowding declined moderately with the severe drought, yet both remained above values recorded in wild microhabitats even under regular climate; drought effects on crowding, however, appeared to be particularly negative in dwellings (Fig 5). Thus, our results suggest that synanthropic *T*. *brasiliensis* populations (i) were as common as, yet larger than, their wild counterparts under regular climate, and (ii) also fared overall better than wild populations during the 2012–2016 severe drought. These findings are in line with observations that *T*. *dimidiata* dwelling-breeding colonies remained largely stable after an extreme-climate event, hurricane Isidore, struck the Yucatán peninsula in 2002, yet house-invasion by adult bugs transiently increased–thus suggesting that the bugs were flying from heavily disturbed wild habitats (which, however, were not investigated in that study) towards the more stable or buffered man-made habitats [53].

We found that rock microhabitats were often infested by *T*. *brasiliensis* before the drought, with moderate bug density and crowding, and that none of those metrics had changed much at the end of the five-year severe drought (Table 2, Figs 4 and 5). Thus, we found no evidence that rock-microhabitat pre-drought infestation had declined, and model-based estimates suggest moderate reductions of density and crowding that were most likely similar across microhabitat types (Tables 4–9, Figs 4 and 5). These results clearly suggest that extreme drought had, if anything, a modest impact on wild *T*. *brasiliensis* populations occupying their primary rocky habitat–the frequency of infestation did not change and the size of the colonies declined only slightly-to-moderately.

*Pilosocereus* sp. shrubby cacti sampled right after the onset of the severe drought (Fig 3) were often occupied by *T*. *brasiliensis*, yet both density and crowding were already apparently lower than in rocks and dwellings (Table 2, Figs 4 and 5). We found that, when sampled during the last months of the drought, cacti in the same area were much less likely to be infested and supported a much less dense bug population (Tables 2–7, Figs 4 and 5). Crowding, however, either did not change or perhaps even increased slightly in the very few cacti that were infested during the drought (Tables 2, 8 and 9, Figs 4 and 5, S1 Fig). This suggests that most cacti were so poor-quality, as overall expected from secondary habitat, that their *T*. *brasiliensis* populations either went locally extinct with the drought or became so small as to be undetectable. On the other hand, our analyses suggest that relatively large populations managed to survive through the drought in a few cacti–which may, therefore, represent fairly high-quality habitat (Figs 1 and 2). Our data show that bug-catch per unit effort was > 2.0 in three cacti sampled during the drought and in two sampled before the drought, yet infestation was > 10 times higher before than during the drought (Table 2, Fig 4 and S1 Fig).

In sum, our data and analyses suggest, taken together, that most of the dwellings we investigated were top-quality habitat for *T*. *brasiliensis*, with bug populations remaining largely stable through an extreme-drought event. In the wild, most rocks were probably high-quality habitat, with pre-drought density and crowding lower than in dwellings but higher than in cacti–and with the drought having, at most, modest effects on those populations. Most cacti, in contrast, were probably low-quality habitat for wild *T*. *brasiliensis*–low enough as to be apparently unable to sustain bug populations during severe drought. Some bugs, however, survived under such extreme conditions in a few cacti that possibly had the defining features of high-quality microhabitats–adequate physical substrate, adequate microclimate, and adequate blood supply. These hypothesis-driven findings are based on a unique, large dataset and on a thorough analytical strategy that accounted for the particular error distribution of each dependent variable, adjusted for unequal sampling effort and non-independence of spatially-clustered observations, and incorporated model-selection uncertainty via model averaging. There are, however, several important caveats that should be kept in mind when interpreting our results. First, in this report we do not address why some specific microhabitats were of higher quality than others. Instead, we assume (i) that physical substrate, as suggested by, e.g., refs. [21–23,25,31,32,35], was minimally adequate for *T*. *brasiliensis* in most of the microhabitats we sampled, and did not change much with the drought; (ii) that the already dry pre-drought microclimate was overall adequate, as suggested by high infestation frequency and by previous studies on *T*. *brasiliensis* [18–20] and other triatomines [54], and changed more during the drought in poorer-quality microhabitats such as small cacti or less-protected rocky habitats; and (iii) that the blood supply, as suggested by our extensive field observations, was larger in dwellings than in wild microhabitats–and changed less with the drought (a) in dwellings (where people plus domestic and synanthropic vertebrates remained available) than in rock microhabitats (where wild rodents and other small mammals, birds, reptiles, or free-ranging goats were common even during the drought) and (b) in rock microhabitats than in cacti (where wild rodent populations visibly declined during the drought). A detailed assessment of physical-substrate and host-availability effects on wild *T*. *brasiliensis* populations is ongoing and will be the topic of a future report. Second, and along the same lines, we concentrated our sampling on microhabitats that, based on prior experience, we believed could sustain at least small *T*. *brasiliensis* populations. For example, we did not sample small or arboreal (*vs*. creeping) cacti, small isolated boulder outcroppings, dwellings that were of a higher-than-average standard [55] or had been sprayed with insecticides less than about one year before fieldwork, or trees or palms [23,28–30]. Our conclusions, therefore, refer to microhabitats that were considered to be, *a priori*, minimally suitable for *T*. *brasiliensis*, and we make no claims as to how the drought might have affected populations occupying *a priori* less suitable microhabitats–although we would expect particularly strong, negative drought effects. Finally, we note that our analyses assume that any particular microhabitat in which we found no *T*. *brasiliensis* was truly unoccupied; this assumption, however, is probably unrealistic in man-made [32,34] and cactus microhabitats [28], and remains untested in rock microhabitats–a topic we also plan to address in future work. Microhabitats in which we caught no bugs, therefore, may have been either uninfested or occupied by very small populations that went undetected. Because a local population so small as to go undetected is likely to be on the brink of extinction [11,12,50–52], frequent non-detections may also signal a negative effect of microhabitat type, drought, or their interaction. We note, in addition, that adjusting for bug-catch effort and cluster-level dependencies made our results more directly comparable across microhabitat types and climate periods. In sum, we are confident that our study, with its strengths and limitations, provides a reliable (and original) appraisal of the key predictions of the microhabitat-quality hierarchy hypothesis.

### Conclusions and outlook

*Triatoma brasiliensis* populations fared overall better in dwellings and rocky outcrops than in cacti during a five-year extreme drought. Estimates of microhabitat and drought effects on infestation, density, and crowding suggest that only a few cacti, *vs*. many rocks and dwellings, represent good-quality habitat for the species under such extremely harsh conditions. Thus, and as predicted by the microhabitat-quality hierarchy hypothesis, *T*. *brasiliensis* microhabitats appear to lie along a ‘quality gradient’ that runs from top-quality rural dwellings through high-quality, primary rocky habitats and to the lower-quality, secondary habitat represented by shrubby cacti–a few of which, however, are probably high-quality. What specific traits determine, and to what extent, microhabitat quality for *T*. *brasiliensis* remains unclear. Limited data suggest that, if minimal physical-substrate and microclimate requirements are met, vertebrate-host availability may be key to microhabitat quality in both dwellings [32] and cacti [28]; this might, then, also be the case for rock microhabitats. Ascertaining the relative roles of physical substrate, microclimate, and host availability on triatomine-bug microhabitat quality may help us understand whether and how environmental management could synergize insecticide-based control of Chagas disease vectors (see, e.g., ref. [56] and references therein). At any rate, the results of our hypothesis-driven assessment clearly imply that *T*. *brasiliensis* can endure extreme climate by exploiting high-quality microhabitats, whether wild or man-made, in the semiarid Caatinga. This provides a hint of the species’ potential to adapt to the widespread increase in aridity expected under climate change across northern South America [57]. We anticipate that, as long as substandard dwellings remain available to the bugs in the semiarid Brazilian north-east [55], *T*. *brasiliensis* will keep using them as high-quality microhabitats in which to survive and breed–and will likely do so even under extreme climate.

## Supporting information

S1 Dataset Raw dataset. (XLSX)

S1 Fig Bug-catch per unit effort in each microhabitat and period. Black bars highlight microhabitat where no bugs were caught. The insets in the right-hand-side panels show cacti with non-zero bug catches, which are barely visible, given the scale of the *y*-axes, in the main graphs.
(TIFF)
